# Improving MiniHip femoral prosthesis positioning using a cross‐laser projection system in total hip arthroplasty by an anterolateral supine approach

**DOI:** 10.1002/rcs.2214

**Published:** 2021-01-09

**Authors:** Hideki Fujii, Tetsuo Hayama, Toshiomi Abe, Motoi Takahashi, Yohei Matsushita, Ryuichi Sato, Takuya Otani, Mitsuru Saito

**Affiliations:** ^1^ Department of Orthopaedic Surgery The Jikei University School of Medicine Tokyo Japan

**Keywords:** anterolateral supine approach, hip arthroplasty, MiniHip, minimally invasive surgery, patient‐specific, short stem

## Abstract

**Background:**

The authors developed a cross‐laser projection system (CLP) to place a femoral neck‐sparing short stem using the minimally invasive anterolateral supine approach in total hip arthroplasty. This study aimed to verify the utility of CLP.

**Methods:**

Thirty joints were assessed with the MiniHip (Corin). The authors compared femoral component implantation with a patient‐specific femoral osteotomy guide (PSG) for the femoral neck‐cut (PSG group), with the CLP attached to the rasp handle to irradiate the cross‐laser to the target of PSG (CLP group), and without PSG or CLP (control group).

**Results:**

In the CLP group, the positional deviation of anteversion, anterior/posterior tilt and varus/valgus placement of the stem postoperatively were 1.8° ± 0.2°, 2.0° ± 2.0° and 2.0° ± 0.1°, respectively. The positional deviation of anteversion (*p* < 0.001) and anterior/posterior tilt (*p* = 0.036) were significantly smaller than those in the other groups.

**Conclusions:**

CLP improves the accuracy of MiniHip femoral prosthesis placement.

## 1 INTRODUCTION

**FIGURE 1:**
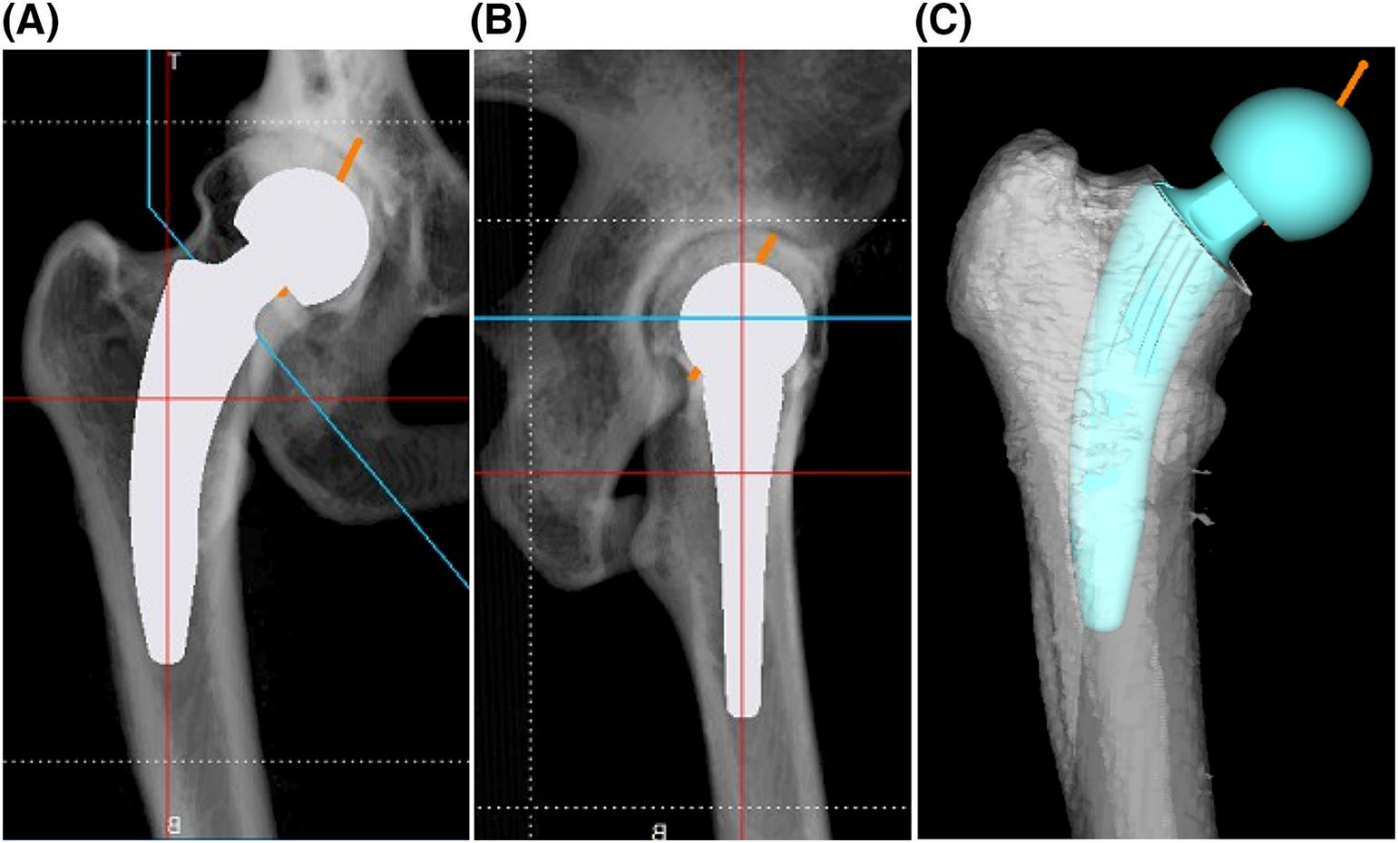
Postoperative radiographs showed varus stem alignment in the anteroposterior view (A) and anterior tilt stem alignment in the lateral view (B). Preoperative three‐dimensional image, anterior view: The MiniHip makes contact with the medial bone cortex proximal to the medullary cavity of the femoral neck and with the lateral bony cortex at the lesser trochanter (C). Owing to the short length and high degree of freedom of placement, the MiniHip prosthesis can reproduce the true centre of the femoral head. A guidance system for positioning the femoral neck‐cut and the angle of insertion of the stem will be useful

For load transmission physiologically proximal to the femur, various short stems have been developed and used in clinical practice. Short stems have a higher degree of freedom of placement than conventional classical stems due to their size, and therefore, advanced techniques are required for short stem placement according to the preoperative plan.^1^, ^2^, ^3^ The standard‐length stem inserted along the femoral metaphysis requires attention only to the height of placement and anteversion. However, short stems also require attention to stem tilt: anterior/posterior and varus/valgus, depending on their length. For placement of a femoral neck‐sparing short stem such as MiniHip prosthesis (Corin) according to its design concept, appropriate surgical techniques and methods that enable accurate reproduction of the three‐dimensional (3D) preoperative plan are required (Figure 1).

In recent years, a minimally invasive surgical approach that avoids damage or injury to the muscles and tendons has been recommended for early recovery. The anterolateral supine approach (ALS) is a surgical method without myotomy or tenotomy; it has high resistance to dislocation after total hip arthroplasty (THA) but restricts the operative field.^4^, ^5^, ^6^, ^7^ In minimally invasive ALS, short stems are easy to handle because of their length, but they are difficult to place accurately. Therefore, techniques to prevent incorrect placement are needed.

At present, a computed navigation system has been developed that accurately reproduces preoperative planning intraoperatively.^8^, ^9^, ^10^, ^11^, ^12^, ^13^, ^14^, ^15^ However, this system has not become popular because of high operational costs, prolongation of operative duration, intraoperative problems between the operator and instrument and spatial restriction of additional instruments in the operating room.^16^ In contrast, the patient‐specific femoral osteotomy guide (PSG) has the advantage of being able to prepare a guide tailored for bone morphology specific to patients and is relatively inexpensive. PSG is commercialised for knee joint surgery and is widely used in clinical practice.^17^, ^18^, ^19^ However, it is mainly used as an osteotomy guide, and it has also been reported that there is no superiority in implant placement.^20^ In THA, it was reported that PSG was used on the acetabular side,^21^, ^22^ but in recent years, it has occasionally been reported that there are some advantages to using PSG on the femoral side.^23^, ^24^, ^25^, ^26^, ^27^, ^28^

**FIGURE 2:**
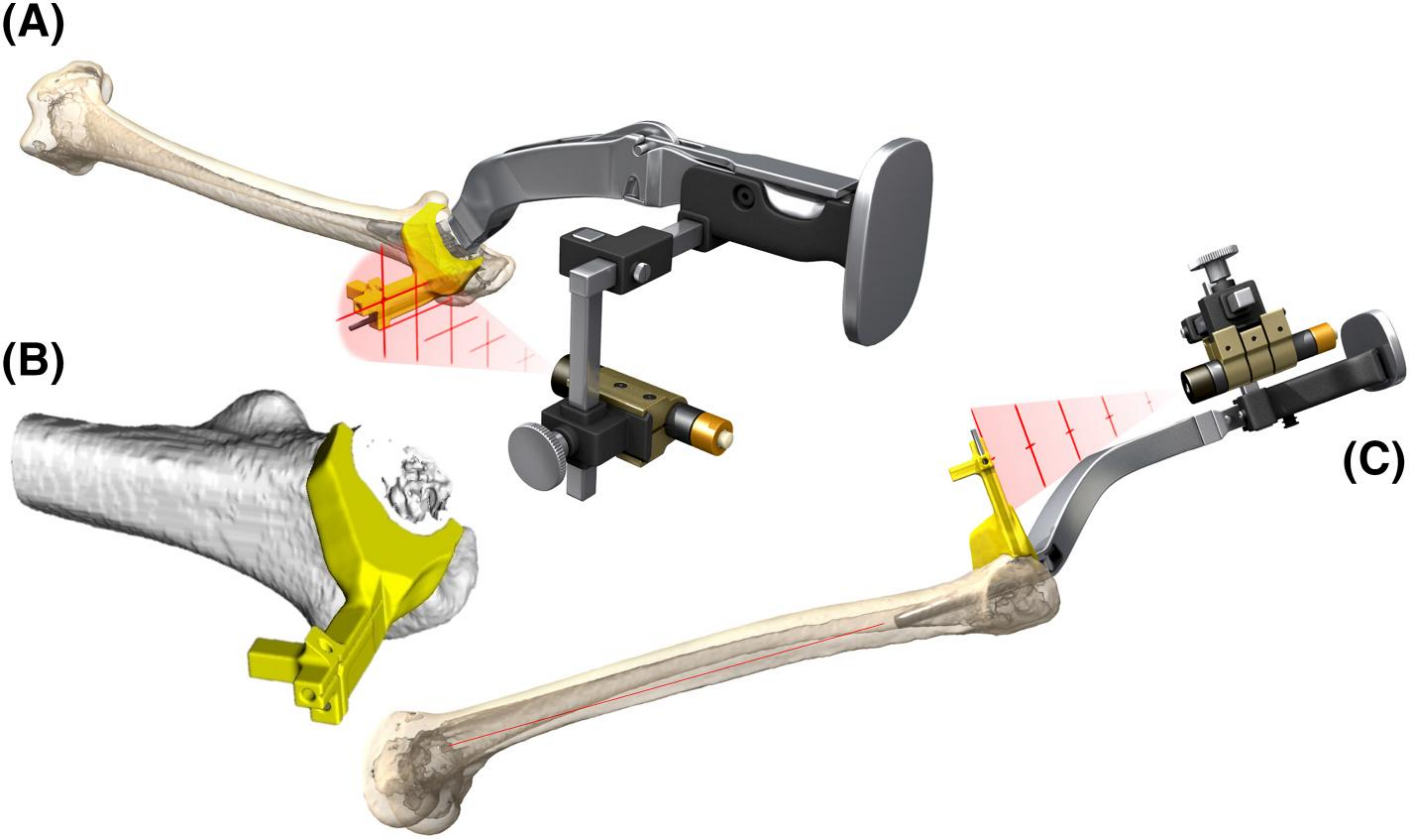
Cross‐laser projection system (CLP): it enables to perform rasping by irradiating the cross‐laser to the target of PSG while confirming the 3D direction of stem at all times. (A) Schema of the CLP (frontal view); (B) Enlarged view of PSG; (C) Schema of CLP viewed from the side. The stem inclines anteriorly than the bone axis

We experimentally produced the design of PSG as an osteotomy guide, enabling accurate placement of the stem even in the minimally invasive operation by ALS. Moreover, we attempted to place the MiniHip by using a device that irradiates the cross‐laser installed to the rasp handle to the targeted PSG attached to the femoral neck in accordance with the preoperative plan. There have been no reports on the use of stem alignment guides in combination with femoral osteotomy guides for accurate placement of the short stem in minimally invasive ALS. We named this device the cross‐laser projection system (CLP; Figure 2). The objective of this study was to verify the utility of CLP.

## 2 METHODS

We assessed 30 joints on which THA was performed by minimally invasive ALS using MiniHip from July 2015 to May 2019. The mean age was 52 years; there were 5 males and 25 females, and the average body mass index was 23 kg/m^2^ (Table 1).

**TABLE 1:** Demographics

AVE ± SD	CLP (*n* = 11)	PSG (*n* = 11)	Control (*n* = 8)	*p* value for all
Gender (F:M)	11 : 0	8 : 3	6 : 2	0.214^a^
Age at surgery (years)	51.7 ± 3.4	51.8 ± 3.9	51.9 ± 3.8	0.996^b^
BMI (kg/m^2^)	23.1 ± 4.0	23.1 ± 5.1	22.9 ± 3.3	0.994^b^
Surgery side (R:L)	7:4	8:3	3:5	0.325^a^
Diagnosis (OA:ION)	7:4	11:0	7:1	0.055^a^

*Note*: *n*, mean ± SD.

Abbreviations: BMI, body mass index; CLP, cross‐laser projection; ION, idiopathic osteonecrosis; OA, osteoarthritis; PSG, patient‐specific femoral osteotomy guide.

^a^ Fisher's exact test (for all).

^b^ One‐way analysis of variance.

### 2.1 Preoperative planning

Using the computed tomography (CT) data obtained preoperatively, preoperative planning was performed with 3D software (ZedHip, Lexi). The patient's DICOM data were read and displayed in axial, sagittal and coronal views to review the images and perform 3D placement positions, including stem and cup size selection. The MiniHip was placed in contact with the medial bone cortex proximal to the medullary cavity of the femoral neck and with the lateral bony cortex at the lesser trochanter on the anterior view, and tilts more anteriorly than does the femoral bone axis along the neck on the lateral view (Figure 1).

### 2.2 Production of the PSG

**FIGURE 3:**
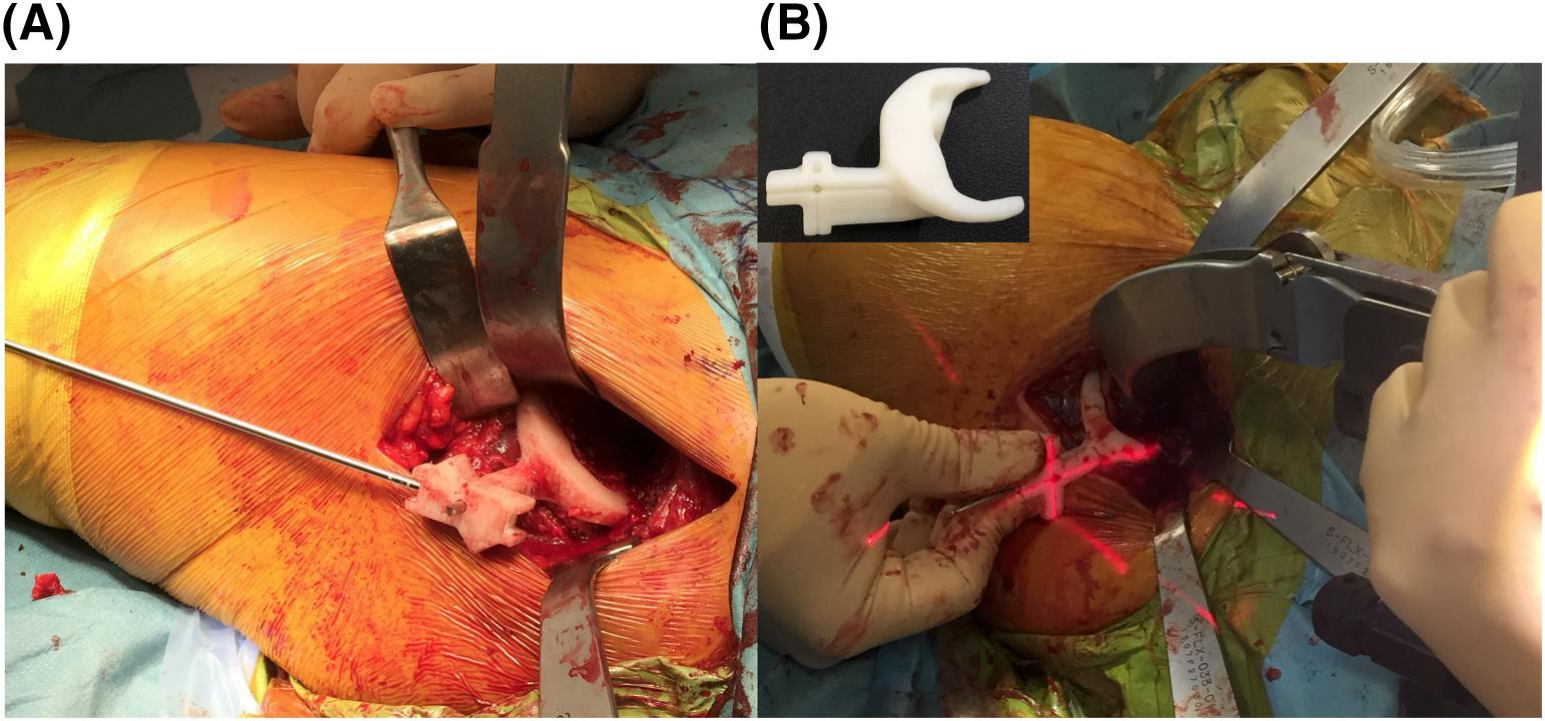
(A) The intraoperative procedure of rasping the femoral canal as guided by the Kirschner wires. (B) Using a cross‐laser projection system (CLP), the cross‐laser is irradiated to the target of the PSG accurately

The PSG was designed for use in preoperative 3D planning, via computer‐aided design software (Solidworks; Dassault Systèmes SA) and 3D modelling software (Geomagic Freeform; 3D Systems). The PSG consists of a base part, which overlaps the surface of part of the femoral neck, and a guide part that is combined with the base part, which has holes to allow the insertion of 2‐mm Kirschner wires. Ito et al.^26^ created a PSG for a posterior approach to the hip joint, and we modified the PSG to approach the hip joint anteriorly for minimal invasion (Figure 3A).

### 2.3 Development of CLP system

We placed a cross target on the PSG and guided the direction of the stem three dimensionally by irradiating the cross‐laser beam installed to the rasp handle to this target (Figures 2 and 3B). We named this device CLP. We developed this device uniquely in cooperation with ArthroDesign. We filed a patent application for this technique, which was accepted (patent application no. 2018‐237311, reference no. ARTHRO International Patent Classification A61F 2/46, identification no. 510183475, submitted on 19 December 2018).

### 2.4 Surgical technique

In 11 joints in the PSG group, osteotomy was performed while the PSG was in close contact with the anterior surface of the femoral neck. Using this osteotomy surface and the 2‐mm K wire attached to the PSG as indices, the stem was placed (Figure 3A). In 11 joints in the CLP group, the CLP cross‐laser was installed on the rasp handle and was irradiated to the target of the PSG attached to the femoral bone side. By matching the cross‐laser to the cross‐target, the stem anteversion, anterior/posterior tilt and varus/valgus were determined, and the actual object was placed after placement of the final rasp (Figures 2 and 3B). In eight joints in the control group, the stem was placed on a freehand without using PSG and CLP.

All operations were performed under general anaesthesia using an ALS. The interval between the tensor fasciae latae and gluteus medius muscles was opened using minimally invasive instruments,^4^ ^,^ ^5^ and the operations were performed by the author in a single institution. The MiniHip femoral component was used in all cases. The acetabular cup used in this study was the Trinity cup (Corin) in all cases. The optimal windows of abduction and anteversion angles of the acetabular cup were 35°–45° and 10°–25°, respectively. Bearing combinations included cobalt‐chrome on polyethylene, and femoral heads were either 28 or 32 mm in size. In addition, all patients received 1000 mg of intravenous tranexamic acid just before surgical incision and just prior to wound closure. Postoperatively, the patients underwent a standard rehabilitation protocol. They were mobilised with the assistance of physical therapy, and full weight‐bearing was allowed with the use of a walker on the first postoperative day.

### 2.5 Postoperative evaluation

All patients underwent a CT scan 1 week postoperatively, and the scans were transferred to the postoperative evaluation software (ZedHip). The authors determined the difference in the stem position (anteversion, anterior/posterior tilt and varus/valgus) between the preoperative planning and the postoperative measurement, with the absolute difference defined as the accuracy of stem placement. The incidence of accuracy outliers was analysed. Surgical data were also recorded, including operative time, intraoperative estimated blood loss, need for reoperation and the presence of complications such as infection, venous thromboembolism and dislocation. All patients were followed up for a minimum of 12 months postoperatively to identify complications.

### 2.6 Statistical analysis

Continuous data are reported as the mean ± SD, if normally distributed, and as the median and interquartile range, if not normally distributed. Categorical data are shown as numbers. Categorical variables were compared using Fisher's exact test. Among‐group comparisons for continuous variables were made by one‐way analysis of variance, if normally distributed and Kruskal–Wallis test, if not normally distributed. Between‐group comparisons for continuous variables were made using the unpaired *t* test and Mann–Whitney *U* test. Multiplicity of comparisons among groups was determined using Bonferroni correction. All statistical analyses were performed using SPSS version 22.0 for Windows (IBM Japan). *p* values less than or equal to 0.05 were considered significant.

### 2.7 Ethics approval and consent to participate

This study was conducted in accordance with the World Medical Association Declaration of Helsinki and was approved by the Institutional Review Board of Jikei University (Approval number: 29‐037(8653)).

## 3 RESULTS

**FIGURE 4:**
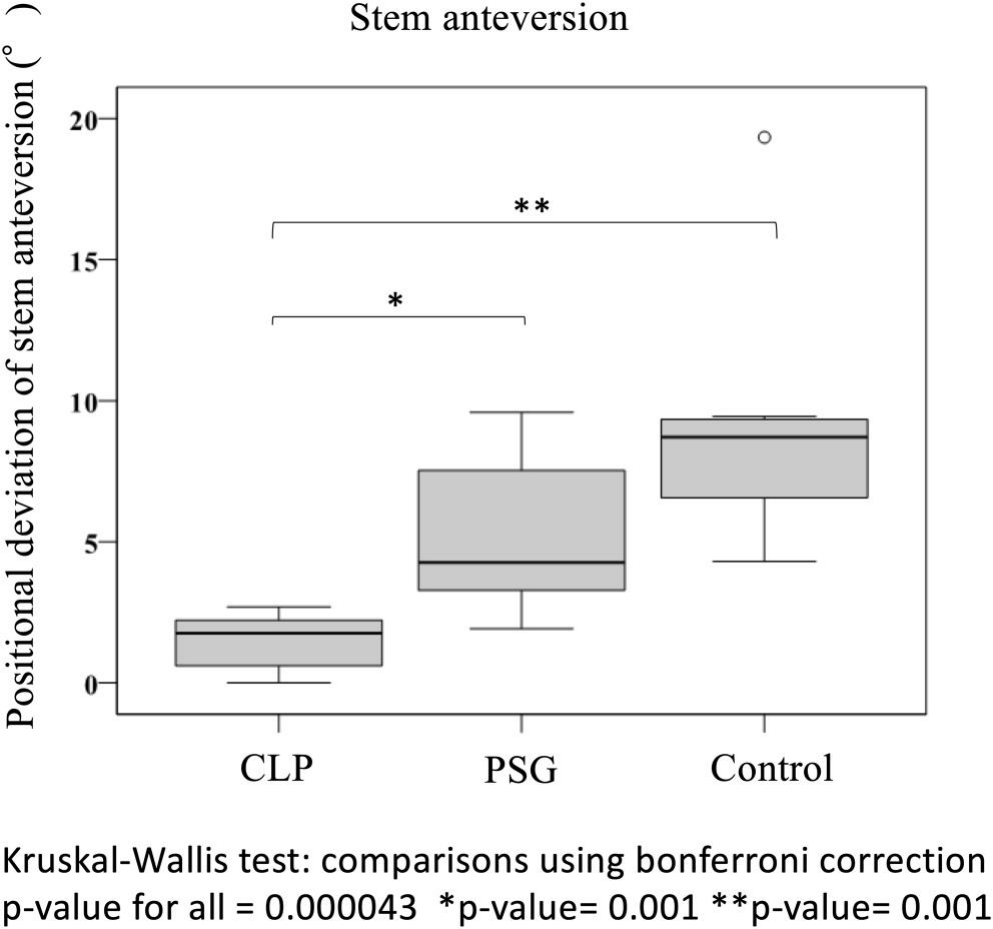
Stem positional deviation between preoperative planning and postoperative evaluation: accuracy of stem anteversion

**FIGURE 5:**
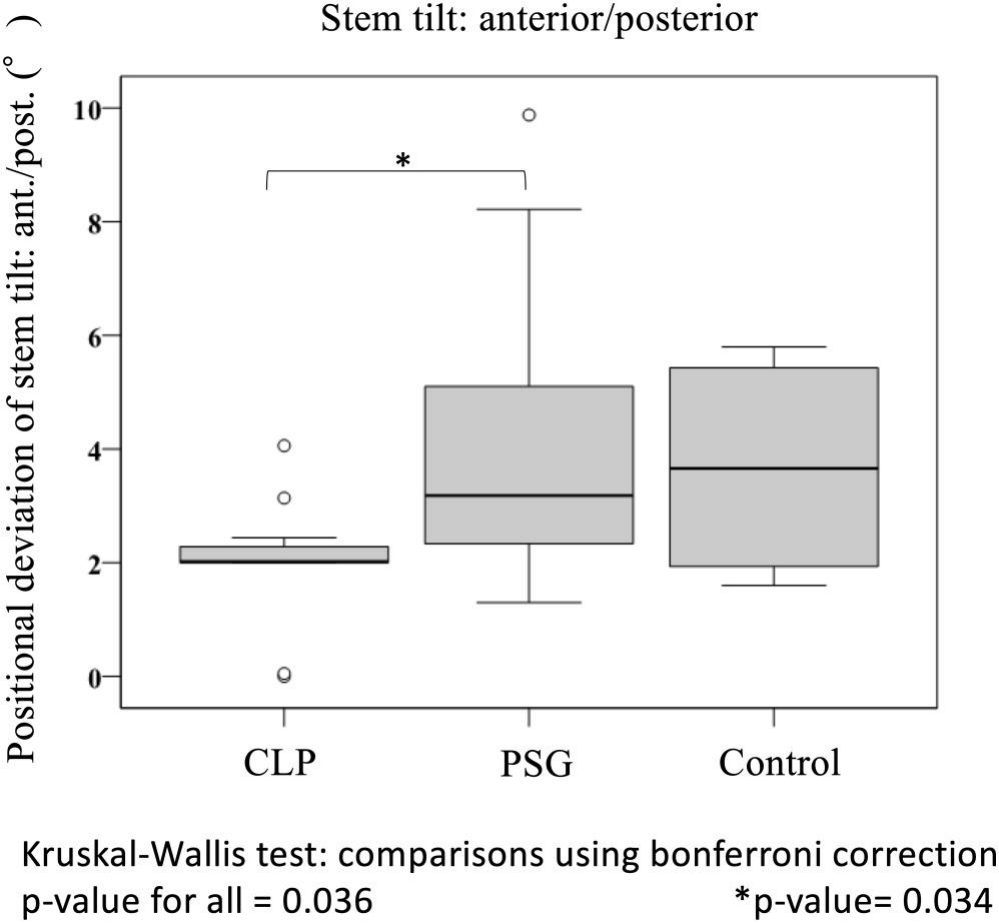
Stem positional deviation between preoperative planning and postoperative evaluation: accuracy of stem anterior/posterior tilt

**FIGURE 6:**
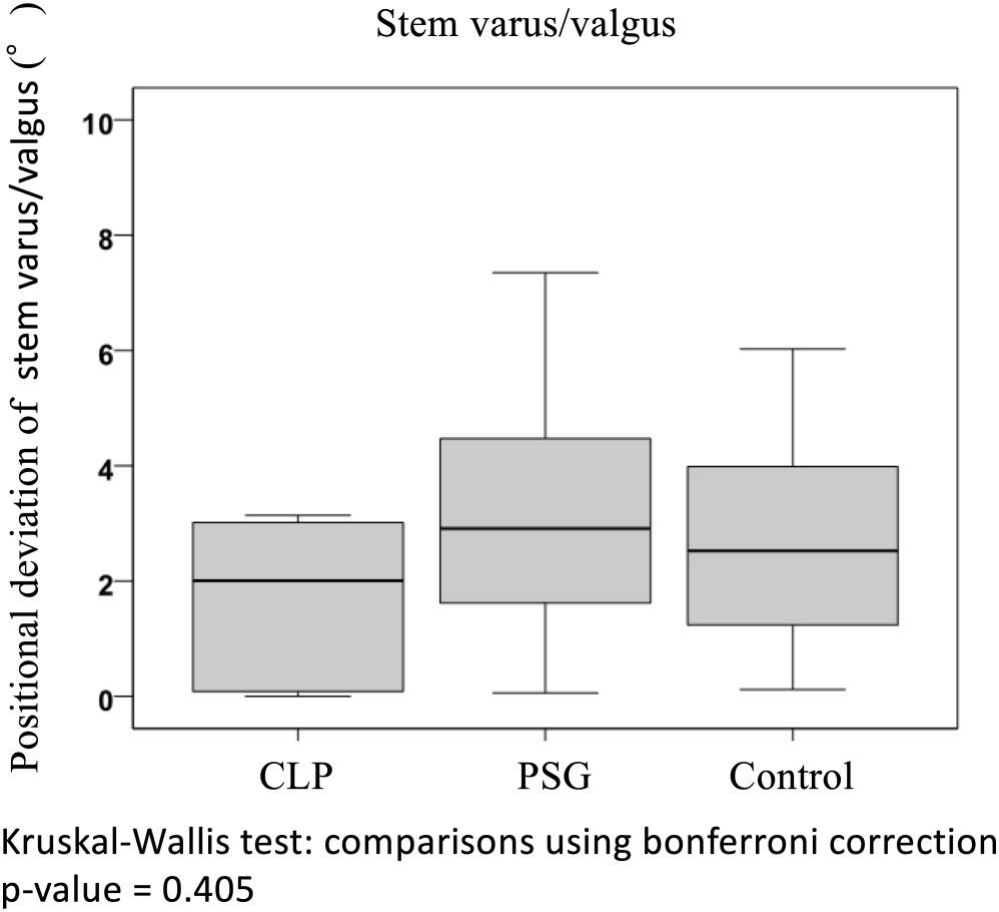
Stem positional deviation between preoperative planning and postoperative evaluation: accuracy of stem varus/valgus

In the PSG group, the positional deviation of anteversion, anterior/posterior tilt and varus/valgus of the stem before and after operation were 4.3° ± 2.9°, 3.2° ± 2.2° and 2.9° ± 1.4°, respectively. They were 1.8° ± 0.2°, 2.0° ± 2.0° and 2.0° ± 0.1° in the CLP group and 8.7° ± 5.9°, 3.7° ± 1.9° and 2.5° ± 1.1° in the control group, respectively. Among the three groups of the CLP, PSG and control groups, there was a significant difference in the positional deviation of anteversion (*p* < 0.001) and anterior/posterior tilt (*p* = 0.036) of the stem (Figure 4). In the CLP group, the positional deviation was significantly smaller for stem anteversion in comparison with the other groups (CLP group vs. PSG group: *p* = 0.001, CLP group vs. control group: *p* < 0.001) (Figure 4). Moreover, in the CLP group, the deviation was significantly smaller for stem anterior/posterior tilt in comparison with the PSG group (*p* = .034) (Figure 5). In all groups, femoral neck‐cut‐level and height of the stem were according to the preoperative 3D plan (Figure 6).

During and after operation, complications such as infection, fracture and dislocation and subsidence and loosening of the stem were not observed. Good excursion and reacquisition of walking ability were achieved in all patients. The Japanese Orthopaedic Association Hip‐Disease Evaluation Questionnaire) was improved from 52 points before surgery to 79 points after operation.

## 4 DISCUSSION

Since the femoral neck‐sparing short stem such as MiniHip prosthesis has the shortest body than the other short stems, the degree of freedom of placement increases. Therefore, it is difficult to place the stem according to the preoperative plan. In ALS, the technique of placing the PSG toon the femoral neck and performing osteotomy using PSG as a guide was considered difficult because the operative field was narrow, but osteotomy could be performed on all 22 joints by devising an appropriate design of PSG. Sakai et al.^27^ also reported that a good osteotomy line could be prepared by preparing a small osteotomy guide using the anterolateral approach in a cadaver. The present study similarly shows that our PSG is useful as a design for the placement of short stems through minimally invasive ALS in actual surgery. Ito et al.^26^ reported that the alignment of the stem of standard length could be controlled using PSG using the posterior approach. When the authors actually used this PSG in ALS, however, there was a possibility of incorrect judgement of placement position and angle in the method in which Kirschner wires were attached to PSG and the alignment of the stem was confirmed visually when checking diagonally. At the time of the actual operation, the long Kirschner wires obstructed the operation, and they were removed. A simple method that does not obstruct the operation is desirable.

The CLP that we developed is able to perform rasping while checking the 3D direction of the stem constantly by irradiating the cross‐laser to the target of the PSG. In the past, PSG has been used for osteotomy of the femoral neck in accordance with the preoperative plan.^25^, ^26^, ^27^, ^28^ A device that can guide the alignment three‐dimensionally at the time of insertion of the stem, such as the CLP, has not been reported so far. Hirata et al.^29^ evaluated neck anteversion during operation using the lower leg shaft as an index and showed that the error of the surgeon in implementing THA was 7.3°. Moreover, Kitada et al.^23^ reported that the accuracy of stem anteversion placement by the CT‐based navigation system was within 5° in 60% of patients. Schneider et al.^28^ performed osteotomy of the femoral neck using the PSG with the direct superior approach and placed stems of standard length. They reported that the accuracy of the height of the osteotomy was within 3 mm.^28^ Sakai et al.^27^ placed an anatomical stem using the PSG by the anterolateral approach using a cadaver. They reported that the verified precision was good. In this study, the precision of implant placement was equal to that reported in these studies. In particular, the stem anteversion was placed very accurately with an average error of 1.8°, which was considered attributable to the system that the cross‐laser corresponds to the target of the PSG. Moreover, the anterior/posterior tilt of the stem was placed accurately. The use of CLP was considered useful in placing the MiniHip by ALS.

## 5 CONCLUSIONS

CLP improves the accuracy of MiniHip femoral prosthesis placement in THA using minimally invasive ALS compared with the procedure without CLP.

## CONFLICT OF INTERESTS

The authors declare that there are no conflict of interests.

## AUTHOR CONTRIBUTIONS

Design of the work, surgeries and manuscript: Hideki Fujii. Design of the work and surgeries: Tetsuo Hayama. Surgeries and data analysis: Toshiomi Abe and Motoi Takahashi. Data acquisition: Yohei Matsushita, Ryuichi Sato and Takuya Otani. Manuscript revision: Mitsuru Saito. All authors have read and approved the final manuscript.

## ETHICS STATEMENT

Informed consent was obtained from each patient.

## Supporting information

Supplementary Material 1

## Data Availability

This study was carried out at the Hospital of the Jikei University School of Medicine (3‐25‐8 Nishi‐Shinbashi Minato‐ku, Tokyo, Japan). The datasets used and/or analysed during the current study are available from the corresponding author upon reasonable request.
